# Supplementary material for: Integrating Proteomics and Metabolomics Approaches to Elucidate the Mechanism of Responses to Combined Stress in the Bell Pepper (Capsicum annuum)
Source: Plants (Basel). 2024 Jul 5;13(13):1861. doi: 10.3390/plants13131861 (PMC11244445; doi:10.3390/plants13131861)
Supplement: Supplementary file 1 [file plants-13-01861-s001.zip › plants-3071501-supplementary/Supplementary Materials/Figure S1.pdf]

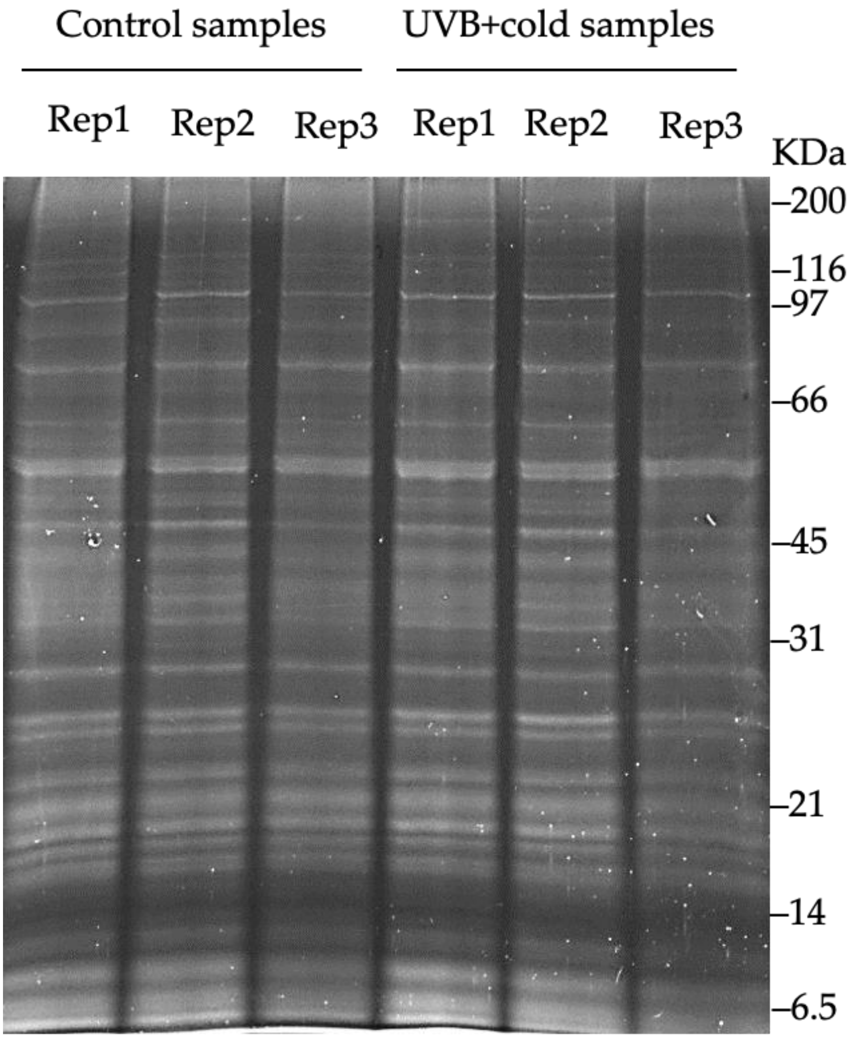


**Figure S1. The protein band pattern in an SDS-PAGE stained with SYPRO-Ruby.** The 200 KDa molecular weight protein marker, is presented on the right side of the gel. Rep1, Rep2, and Rep3 indicate the biological replicates utilized for control and UV-B+cold.
