# Supplementary material for: Integrating Proteomics and Metabolomics Approaches to Elucidate the Mechanism of Responses to Combined Stress in the Bell Pepper (Capsicum annuum)
Source: Plants (Basel). 2024 Jul 5;13(13):1861. doi: 10.3390/plants13131861 (PMC11244445; doi:10.3390/plants13131861)
Supplement: Supplementary file 1 [file plants-13-01861-s001.zip › plants-3071501-supplementary/Supplementary Materials/Figure S2.pdf]

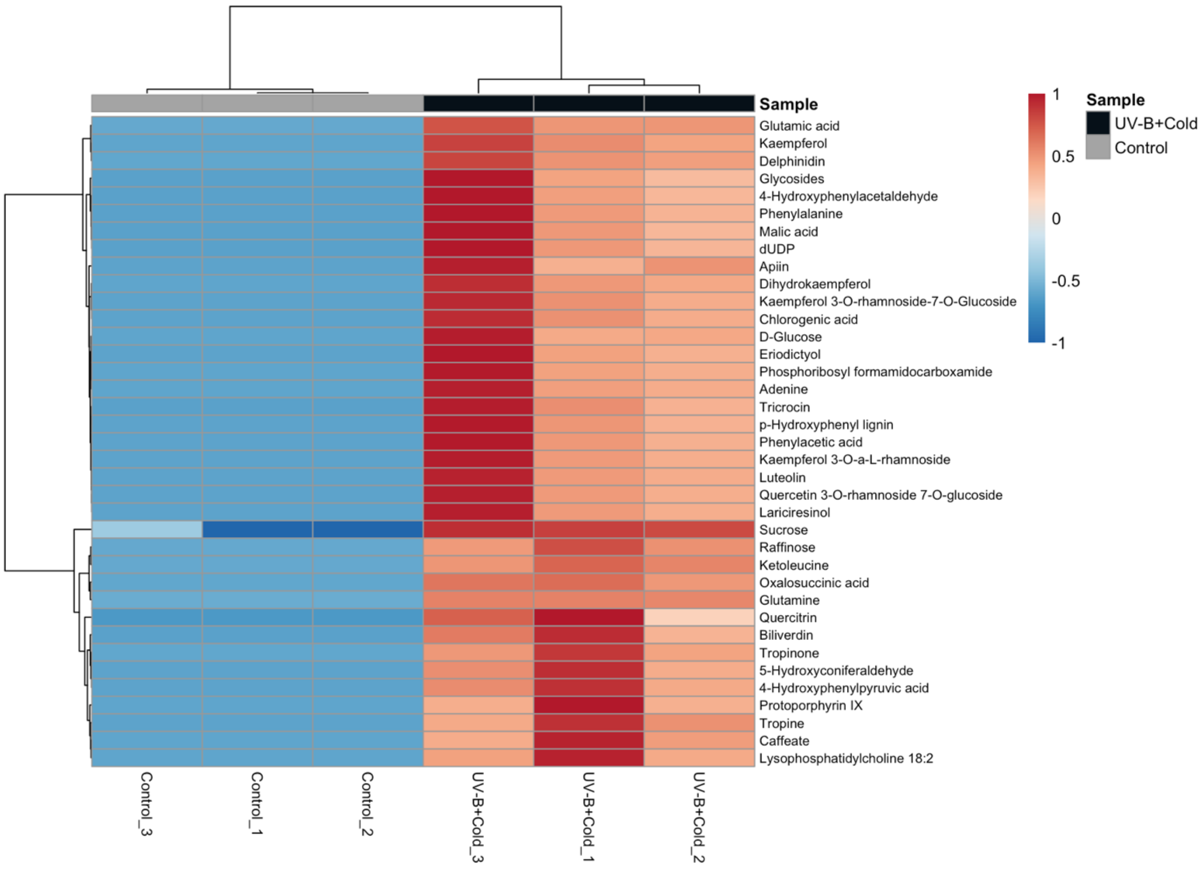

Figure S2. The heatmap analysis shows the variation in metabolite abundance in the stems of bell pepper plants in the control (gray) and UV-B+Cold (black) samples. The colors blue to red represent the intensity normalization value (data transformation: Log10 Normalization; data scaling: Pareto Scaling). Ward's algorithm uses hierarchical clustering to separate individual samples (x axis) and compounds (y axis) and scales the dendrogram to represent the distance between each branch (distance measure: Euclidean).
