# Supplementary material for: Integrating Proteomics and Metabolomics Approaches to Elucidate the Mechanism of Responses to Combined Stress in the Bell Pepper (Capsicum annuum)
Source: Plants (Basel). 2024 Jul 5;13(13):1861. doi: 10.3390/plants13131861 (PMC11244445; doi:10.3390/plants13131861)
Supplement: Supplementary file 1 [file plants-13-01861-s001.zip › plants-3071501-supplementary/Supplementary Materials/Figure S3.pdf]

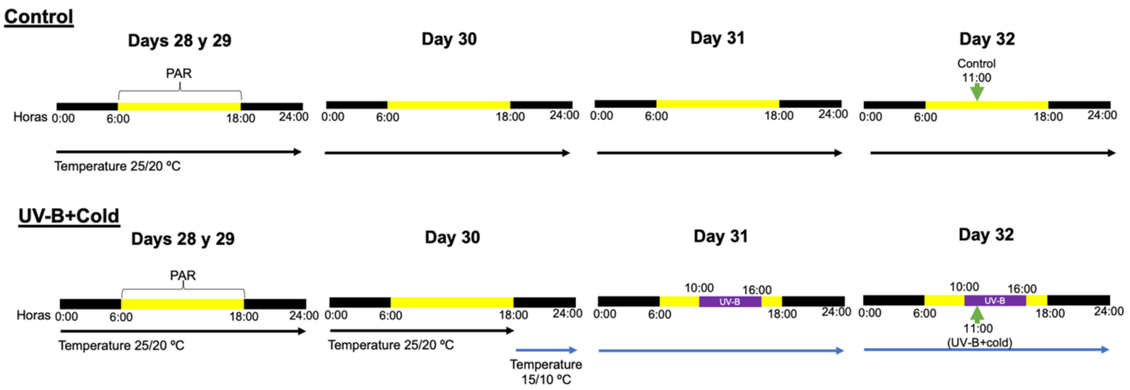

Figure S2. The scheme illustrates the experiment's design to produce bell pepper seedlings in both control and UV-B+cold conditions. The green arrow indicates the samples collected. The black box shows the times without light. The yellow box represents the PAR radiation. The purple boxes indicate the UV-B radiation (72 kJ m^2) for 6 h.
